# Supplementary material for: Splice-Junction-Based Mapping of Alternative Isoforms in the Human Proteome
Source: Cell Rep. Author manuscript; Available in PMC 2020 Jan 15. (PMC6961840; doi:10.1016/j.celrep.2019.11.026)
Supplement: 2 [file NIHMS1546469-supplement-2.zip › DF1/PXD006675/RightAtrium/RightAtrium_2_SVIL_RPELCTSHSETPTVDDEEK.pdf]

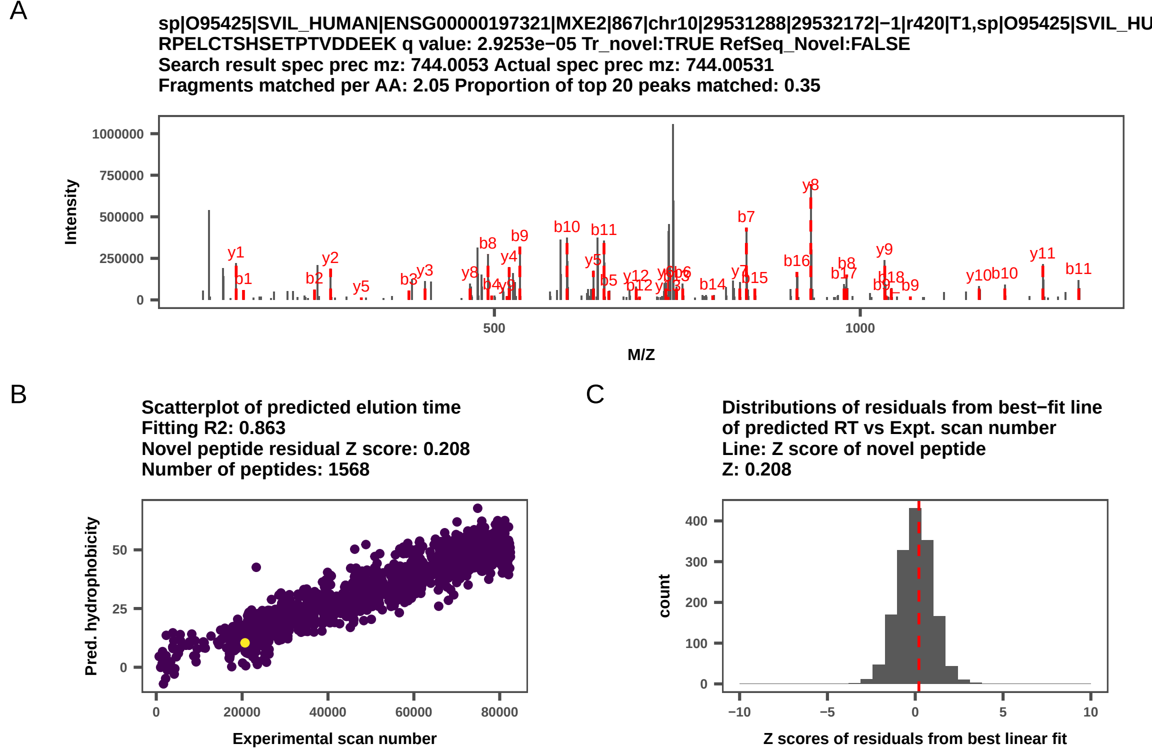
A
sp|O95425|SVIL_HUMAN|ENSG00000197321|MXE2|867|chr10|29531288|29532172|−1|r420|T1,sp|O95425|SVIL_HU
RPELCTSHSETPTVDDEEK q value: 2.9253e−05 Tr_novel:TRUE RefSeq_Novel:FALSE
Search result spec prec mz: 744.0053 Actual spec prec mz: 744.00531
Fragments matched per AA: 2.05 Proportion of top 20 peaks matched: 0.35
Intensity
1000000
750000
500000
250000
0
500
1000
M/Z
y1
b1
b2
y2
y5
b3
y3
y8
b8
b4
y4
y9
b9
b10
y5
b11
b5
y12
b12
b14
b7
y7
b15
b16
y8
b8
b17
y9
b18
b9
y10
b10
y11
b11
B
Scatterplot of predicted elution time
Fitting R2: 0.863
Novel peptide residual Z score: 0.208
Number of peptides: 1568
Pred. hydrophobicity
50
25
0
0
20000
40000
60000
80000
Experimental scan number
C
Distributions of residuals from best−fit line
of predicted RT vs Expt. scan number
Line: Z score of novel peptide
Z: 0.208
count
400
300
200
100
0
−10
−5
0
5
10
Z scores of residuals from best linear fit
